# Innovation indices: the need for positioning them where they properly belong

**DOI:** 10.1007/s11192-015-1632-4

**Published:** 2015-07-07

**Authors:** Jan Kozłowski

**Affiliations:** Ministry of Science and Higher Education, Poland, Wspolna 1/3, 00-529 Warsaw, Poland

**Keywords:** Index, Scoreboard, Innovation, Innovation studies, Innovation policy, Self-fulfilling prophecy, System approach, Big data

## Abstract

A specific quality of the discussion about innovation indices (scoreboards) is that more often than not the subject is dealt with from a purely technical point of view. Such a narrow approach silently assumes that indices used as a policy tool are an accurate reflection of the phenomenon and should not be questioned, and also that the whole discussion concerning them should refer to methodological aspects and is best left to the statisticians. This author is of the opinion that for an accurate evaluation of the value of indices as a policy tool, it is necessary to consider the matter from the broader point of view and from the context in which such indices are generated and used. This article puts forward the thesis that progress in science and innovation policy studies depends on a diversity of issues, approaches and perspectives. If that is the case, maintaining thematic and methodological variety may be more important than creating coherent and closed analytical tools, i.e. indices. The advantage of indices is that they focus attention on those variables which are deemed to be key. Among their disadvantages, however, are their highly abstract nature (in order to understand innovation-related phenomena, it is necessary to study them in tangible, composite forms); their tendency to skip unmeasurable determinants; their prior acceptance of definitions and concepts of innovation (instead of searching for them); the way they apply a single yardstick to diverse countries and regions, assumed linearity and causality in a complex and non-linear world, the way they direct policy towards implementing indicators (rather than identifying and solving problems). It is suggested that big data revolution will allow the emergence of a new measurement tools that will replace innovation indices.

## Introduction

“Innovation” and “indicators” reign everywhere today and they seem to be a symptom of modernity.^1^ No matter whether we use Google, Ngram Viewer, WorldCat, Web of Science or media content data bases as a measure, we encounter an enormous and growing number of entries: e.g. 148,702 items for innovation(s) and 511,200 items for indicator(s) in the titles and sub-titles of printed and electronic materials registered in the WorldCat. It will not be an exaggeration to say that innovation and indicators govern our consciousness. In relation to the immense mental pressure of these concepts, presented in texts, diagrams and models, we remain largely helpless, because they are not sufficiently subject to critical reflection.

During the last decade, approximately 150 innovation indices (a broad term that usually also includes scoreboards, composite indicators, rankings and indicator’s reports) have come into being, put forward by international organisations, think tanks, universities or individual researchers. Many of these emerged at the project stage, and while some have only been produced on one or few occasions, many continue to be produced. One of the best-known is the EU’s *Innovation Union Scoreboard.* The popularity of innovation indices goes hand in hand with the popularity of innovation surveys, which are systematically carried out in c. 80 countries (OECD 2014a). Often innovation indices derive indicators from the surveys (e.g. Innovation Union Scoreboard from Community Innovation Survey). They are used as diagnostic tools for identifying problems and needs, measures of performance, techniques of awareness-raising and public advocacy and instruments of change (Davis and Kingsbury 2012) and a basis for benchmarking innovation systems and innovation policy at the national, regional and city level. As a rule, they refer to areas of significance for research and innovation policy, such as innovation, entrepreneurship, R&D, technology, creativity, science-industry links, education, intellectual capital, and ICT.

Innovation indices are described and discussed mainly by their creators and users, from a statistical perspective. As an example we can cite the chapter on scoreboards in the influential *Handbook of Innovation Indicators and Measurement* (Hollander and Janz 2013). A specific quality of the discussion about innovation indices (scoreboards) is that more often than not the subject is dealt with from a purely technical point of view. Such a narrow approach silently assumes that indices used as a policy tool are an accurate reflection of the phenomenon and should not be questioned, and also that the whole discussion concerning them should refer to methodological aspects and is best left to the statisticians. This author is of the opinion that for an accurate evaluation of the value of indices as a policy tool, it is necessary to consider the matter from the broader point of view and from the context in which such indices are generated and used.

The statistical perspective assumes a particular position concerning the relationship of statistics to reality: realism. Statisticians (data collectors and analysts) assume that measures capture more or less accurately some feature of an external world. However, others, in particular sociologists, anthropologists and science-studies scholars, understand “the objects targeted by measurement as products of measurement and measurement conventions that are negotiated and variable” (Desrosieres 2001; Espeland and Stevens 2008). This attitude is called social constructionism, seen as a part of the anti-positivist approach. As Lazarsfeld and Barton said years ago: “[B]efore we can investigate the presence or absence of some attribute… or before we can rank objects or measure them in terms of some variable, *we must form the concept of that variable* (Lazarsfeld and Barton 1951 cited after Goertz 2005).

The social constructionism attitude radically changed the image of indices as a subject of research. Instead of a “taken for granted” attitude, which at best leads to attempts at improvement, it gives you an opportunity to formulate key questions about their origins, assumptions, validity, construction, use, and consequences. These questions started to be raised quite recently (comp. Davis et al. 2012).

Loosely fitting into the social constructionist program of the science studies (comp. Restivo and Croissant 2005), this paper tries to use its different ideas to assess the institution of the index. However, most of the arguments are taken from statistical publications.

After a brief presentation of the anti-positivist approach and after describing the phenomenon of indices, the two main parts of the paper present a critical approach to the concept of “innovation” and a critical assessment of innovation indices. The last part contains suggestions on how to get out of the situation.

## Anti-positivist trends

Social constructionism examines the development of socially constructed understandings of the world. It assumes that (1) humans rationalize their experience by creating a models of the social world and (2) language is the most essential tool through which they construct reality (Leeds-Hurwitz 2009). Social constructionism is a multi-faceted phenomenon which is part of the broader anti-positivist movement.

The expansion of statistical and formal methods in social sciences clashes with the expansion of approaches inspired by the anti-positivist one. This clash is visible in discussions on indices. These classical products of quantitative research are criticised using arguments rooted in the anti-positivist approach, formulated under the influence of evaluation of their usefulness as a policy tool.

The anti-positivist approach is difficult to describe in just a few sentences. In general terms, it comes down to a denial of positivist propositions regarding the possibility of gaining knowledge that is unequivocal, independent of the circumstances in which it emerges.

For the purpose of our paper, these propositions may be divided into three (interrelated) groups:

- propositions about the lack of neutrality in the language of inquiry,
- propositions about the possibility of achieving ‘the one, definitive’ perspective,
- propositions confirming that the cognitive content is a ‘self-fulfilling prophecy’.

### Proposition of the lack of neutrality in the language of cognition

Propositions on the neutrality of cognition in relation to statistics, regarded as model accuracy, were developed in *Demystifying Social Statistics* (Irvine et al. 1979). The authors argue that the cult of accuracy, associated with the application of statistical methods, has the disadvantage of resulting in feelings of indifference towards qualitative methods and obscures the fact that statistics itself has ‘soft foundations’. Regarded as the model of objectivity, statistics is based on subjective assumptions. Statistics are not *amassed*, but *generated*; its results are not *laws* or *findings*, but *products*. Let us consider, for example, statistics regarding crime, suicides, illness or unemployment. These are generated in particular organisational contexts and assume an interpretation of the phenomena being observed.

### Proposition of the impossibility of attaining a definitive perspective

Bronowski (1978) wrote that none of our explanations can be true and that the truth accessible to us is never definitive, for when conducting research we must cut reality and decide what is or is not significant. In view of the fact that the world constitutes a whole, and that every fact influences another, each cut is just a convenient simplification, leading to disruption, as a result of which we are only able to decode a fraction of propositions about the whole. For this reason, no result of decoding will ever be true.

### Propositions confirming that cognitive content is a self-fulfilling prophecy

Ferraro et al. (2005) stress that theories shape institutional projects and management practices and also expectations concerning behaviour, in this way creating predictable behaviours. Theory is also immortalized by its propagating language and assumptions which are broadly applied and accepted, treated as clear and self-evident. This is particularly so when a theory, and also concepts, findings, indices etc. enjoy official support.

While these thesis have been the ‘silent truth’ in social sciences and popular consciousness, particularly since the 1980, they have not been operationalized to an extent that would enable a more in-depth evaluation of the costs and consequences of concepts, statements, indicators, indices, conceptual frameworks and theories in the social sciences (comp. Ferraro et al. 2005).

## A glut of indices

The need to process huge amounts of statistical data on research and innovation as a source of economic growth and prosperity into a simple message underpins the modern-day boom in innovation indices.

The concept of an “indicator” concerns: (a) the relations between two data items (e.g. GDP per capita); (b) statistical data of great significance selected to describe a particular aspect of the social world concisely, measure performance and act as a guide to decision making. Indicators rely on statistical information but are not the same as statistics.

Over the years, indicators were differentiated in terms of forms (structural, input, process, output, outcome, impact) and applications (budget; control; evaluation; implementation; management; milestone; monitoring; policy; planning; programme; project; resource efficiency; roadmap; strategy…).

Indicators depend on simplification. They are often numerical representations of complex phenomena intended to render these more simple and comparable with other complex phenomena also represented numerically. Indicators are typically aimed at policymakers and are intended to be convenient, easy to understand, and easy to use (Davis et al. 2010).

If indicators represent a second-order abstraction and packaging of raw statistical data (Davis et al. 2010), the group of composite indicators, indices, scoreboards and rankings represent third-order.

The last 20 years have seen growing interest in the development and use of these third-order tools to improve the way innovation system features are measured and described. The European Commission is particularly active in this field, having produced a comprehensive system of tools for measuring innovation, including *Innovation Union Scoreboard* and its *Summary Innovation Index, Innovation Indicator, Innovation Union Competetiveness Report*, and also *Research and Innovation Performance in EU Member States and Associated Countries*.

### Index in making

Indicators have rarely been the subject of large-scale scientific research; the lack of coherent conceptual frameworks telling us how to create indices, select the correct variables, weight and aggregate them. Recently, critical view at indices have been presented, among others, by Archibugi et al. (2009), Edquist and Zabala (2009), Zabala-Iturriagagoitia et al. (2007).

Most commonly, when creating an index the first stage is arranging divisions into thematic blocks corresponding to various dimensions and aspects of the phenomena being studied. This division is usually based on patterns existing in social sciences, such as “input–process–product–results–impact”, and also “knowledge creation–transfer and transmission of knowledge–use of knowledge”, or “economy–society–culture–politics”. The next step is to select indicators, which should be easily available, retain their value over time and have comparable weight as factors of innovative activity. Finally, the indicators are subject to statistical treatment aiming to determine their value, completeness and coherence (Sajeva et al. 2005; Arundel and Hollanders 2008). Statistical treatment serves to temper the subjectivity of earlier choices.

All choices (both in the construction of indicators and indices and in the formulation of policies using their recommendations) are basically trade-offs. A ship cannot be nimble and yet also have a huge tonnage. Likewise, an index cannot take all aspects into consideration. It is based on the choice of particular characteristics at the expense of others, of significance in one area at the expense of another. It may be a better measure of economic characteristics in a particular type of economy, yet a worse (or deceptive) one in others. It combines subjective elements while giving the illusion of precision that is expected in quantitative data.

### History

Although indices have become a tool for daily use, since the very beginning they have raised quite justified doubts.

Tracking trends in business and the economy using data sequences or cumulative indices has a long history. Since the 1870s American corporations have systematically accumulated, collated and analysed various kinds of data on company efficiency and economic trends. This data was often presented in the form of diagrams (Yates 1989). Demand emerged for succinct snapshots describing the economic situation in a single of a couple of numbers. It was in this way that such indices as *Babson’s Composite Plot,* first published in the United States before World War 1, came into being. At that time, discussions on this index’s value raised similar arguments for and against indices, in the same way as modern-day discussions’ of the value of composite indicators and scoreboards (Copeland 1915; McDowall 2008). The argument in favour of composite indicators was their conciseness. The argument against was the inherent simplification, and critics of *Babson’s Plot* argued that rather than having a single composite indicator, it is better to spend time analysing a series of well-chosen individual indicators. Advocates of indices countered with the assertion that interpreting indices is far easier than striving to identify common trends in an array of disparate indicators.

### Indices as a form of power

Michael Foucault’s findings and insights recently became used in order to present indicators and indices as a hidden form of power and the instrument of global governance (Davis and Kingsbury 2012; Davis et al. 2010, 2012). As is well known, Foucault challenges the idea that power is wielded by people or groups by way of ‘episodic’ acts of domination or coercion, seeing it instead as dispersed and pervasive. ‘Power is everywhere’ and ‘comes from everywhere’. It is a kind of ‘meta-power’ that pervades society, remaining in constant flux and negotiation. Power is constituted through accepted forms of knowledge, such as science (Gaventa 2003; Foucault 1998).

Belief in statistics as a manifestation of power has been proclaimed for some time. “Economic numbers have come to define the world. Individuals, organizations, and governments assess how they are doing based on what these numbers tell them” (Karabell 2014).

According to Davis and Kingsbury (2012) indices and indicators “represents a form of power to define the way the world is understood”. Calling them a measure of ‘innovation’ “asserts a claim that there is such a phenomenon and that the numerical representation measures it. An indicator may even create the phenomenon it claims to measure, as IQ tests came to define what intelligence is. Labeling this measure an Indicator, Index, Ranking, League Table, etc. implies a claim to knowing and measuring a phenomenon.”

### Indices as a part of broader trends

The burgeoning of indices is seen as part of broader trends: the victorious march of abstraction and objectivization (comp. Giddens 1991), quantification (Espeland and Stevens 2008), reflexive modernity (Archer 2012), accountability (Espeland and Vannebo 2007; Espeland and Stevens 2008), and the scientification of society and socialization of science (Frane 2014).

Abstraction, which is necessary for the development of increasingly complex societies, allows the extrication of social relations from the local context and their rearrangement on abstract scaffolding. The components of situations are divided and subsequently recombined in accordance with time (schedules, timetables), space (maps, plans), hierarchy (organograms, decision trees) or other categories. Thanks to this, the following have become possible: law, organisation, expert fields, machines, games (reflecting real events in a schematic way, for example, chess as symbolic battle exercises), money and cheques.

Objectivisation (description, measurement, judgement and the reasons for a decision), in other words the verification of subjective knowledge by established methods (scientific research, legal procedures, peer review, voting etc.) makes for a more predictable world.

Quantification has become a tool of objectification. This refers to the idea of expressing phenomena in quantitative categories, both in measurement (time and space, quality of work, degree to which goals are achieved, value of goods, knowledge and competences etc.), and also in mathematical and statistical analyses (Porter 1995).

### One family

Innovation indices using composite indicators are so strongly correlated with GDP per capita, on the one hand, and are so strongly correlated with each other, on the other hand, so that they all create a single community. The strength of the (Pearson) correlation varies from 0.47 (IUS-KEI) to 0.96 (MIS-KEI). In more than half of the cases it amounts to more than 0.8!
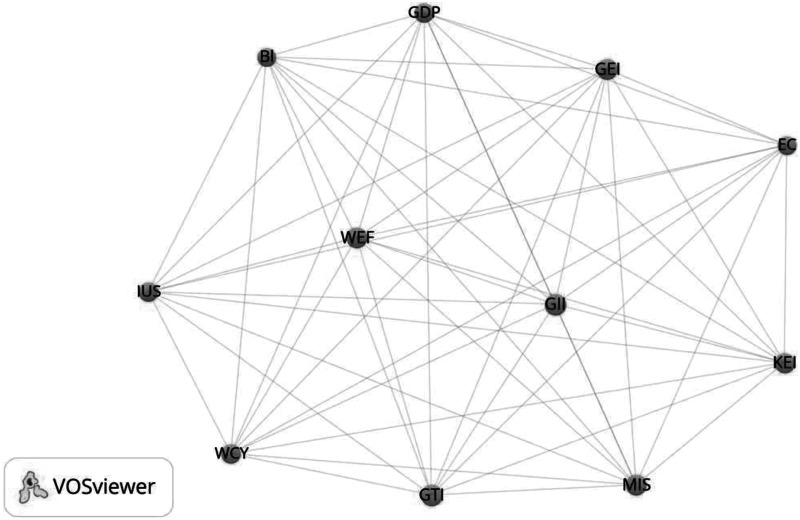

GDP: per capita (the World Bank, 2013, 2012)
WCY: World Competitiveness Yearbook 2014
WEF: Global Competitiveness 2014/2015
GTI: Global Talent Index 2015
GEI: Global Entrepreneurship Index 2015
MIS: Measuring the Information Society Report 2014
Bl: The Bloomberg Innovation Index 2015
IUS: EU Innovation Union Scoreboard 2013
GII: The Global Innovation Index 2015
KEI: Knowledge Economy Index 2012

## Criticism of the concept of innovation

Unlike those researchers who hold that concepts must “fit the world”, social constructionists stressed that concepts are defined relative to simplified mental models of the world.

The category of “innovation” which “appears with a universal claim of validity” (Moldaschl 2010), does not meet the criteria of good social science concept (comp. Gerring 1999, Bjørnskov and Sønderskov 2013).

Especially during the last two decades term lost any limits, become overused to the point of becoming meaningless and is on the fast-track to attain unwelcomed title of “buzzword” (Moldaschl 2010; Shaver 2014). Even if this statement refers to the concept of innovation in general, as it is used in the media and in everyday language, it turns out that it suffers from similar problems also in statistics and research.

### Fuzzy concept

There are many reasons for the recent status of the concept.

One is theoretical. Although innovation studies have already been established and institutionalized at universities and national research agencies, there are no innovation theories, at best, there are conceptual frameworks, such as the National or Sectoral Innovation System. And it is certainly because general innovation theory is hardly possible. Moldaschl (2010) point out, that such a theory would be “as meaningful as a *theory of everything*, at least not if the subject of change that is to be clarified is not in itself homogeneous. Would it then make sense to set up a “theory of the process” without designating a specific class of processes (e.g. biological) relating to the item?”.

Research studies on which innovation policies are based are considered to be too macro and not detailed enough to address the needs of specific regions or sectors (Godin 2013). It is possible that this comes from the difficulties in describing phenomena like innovations—so complex, so varied, understood in so many different ways and prone to sudden change. The existence of such studies would shake the existence of indices, with the *Innovation Union Scoreboard* at the forefront, as these are based on a *universal* concept of innovation and it is difficult to imagine how they could be constructed on the basis of many *microscopic* theories of innovation.

The second reason is empirical. The economic literature on innovation, which mainly explores the correlation between innovation and growth or productivity (at the macro level), or innovation and company’s profits (at micro level), generally states that “innovation matters”. However, it also concludes that the relationship between innovation and economic variables is context-sensitive: at the country, regional or sector level it is contingent upon specific socio-economic characteristics, such as distance from the technological frontier (e.g. Bilbao-Osorio and Rodríguez-Pose 2004; Pessoa), and at the firm level it depends on age of the firm, the type of innovation, and the cultural context (e.g. Rosenbusch et al. 2011).

It is also pointed out, that despite over 50 years of analyses of research and innovation policy, there are still relatively few findings describing “how public investment may lead to a growth in scientific production, an improvement in patenting, increased innovation and may boost national wealth” (Crespi and Geuna 2008).

Experience shows that innovation can be very context and culture-dependent. For example, for European firms there is no innovation when it is not successful, while for American firms innovations that were initially not successful “were not considered failures because they contributed knowledge that could be successfully applied later” (OECD 2014b). Also, the same value of a particular indicator (e.g. new innovations on the market) might have a different meaning in a country such as Austria, where many global companies operate, compared to its meaning in less developed countries.

Although innovations are widely recognised as the engine of economic growth, competitiveness, productivity and employment, measuring them still seems imprecise when compared with the measurement of economic variables such as production, investment, trade and employment. Technological innovation is certainly the most varied economic category, as it refers to products of such diverse technological, economic and social significance as the jet engine or corkscrew. Supplementing technological innovations with those in the fields of organisation and marketing (not to mention in many other non-technological categories) complicates this category still further. It is often difficult (without the benefit of considerable hindsight) to differentiate minor improvements from essential streamlining and even radical innovation. The innovator who is the first to introduce an invention to the market is not in a position to foresee the scale to which it might be diffused, improved and utilised in future (Carter 2007).

The character of innovation is changing rapidly, particularly in economically developed countries, whose competitiveness is founded on innovations. The ‘innovations’ in California in the 1960s, which emerged mainly in the laboratories of large corporations, differ fundamentally from the ‘innovations’ which came into being in the same state half a century later. The latter are ‘open innovations’ in character and more often emerge at universities and in small and medium-sized enterprises.

Finally, whatever the classification of sector we take into consideration, we may state that innovations have different meanings in different sectors. Innovations in the agricultural and food processing sector have a different basis compared to innovations in the electronics, public health, education or finances sectors.

### Meaning imposed in advance

According to Godin, the original sin committed in the literature on innovations, which was transferred to indices and leads to them being devalued, came from their view of innovation as a panacea for all evils, and not from the problem of development in countries for whom innovation is (or is not) the solution. “Innovation is seen as a universal, uncontested and a priori solution rather than tool for resolving the specific problems (needs) of society”. Propagating the idea of innovation in science and culture proceeds on the basis of the fallacious premise that “new” means “better”. This idea is important in commerce (products are advertised as “new” and therefore desirable), not always important in industry, and rarely important in public, or spiritual life.

The words that we use, despite all the attempts at clarification and operationalization, carry the baggage of history.

Until the eighteenth century, an innovator was a suspicious person, one to be mistrusted and innovation was associated with a violation of the divine and social order (Godin 2008). Enlightenment and modernity, on the contrary, and by way of contrast, gave innovators and innovation heroic qualities. According to Frye (1982) a history of language moves through three distinct phases, which he called as metaphor, metonymy, and descriptive phases of language. Without going into Frye’s concept, it should be stressed that earlier phases of language are never completely lost and “shine through” subsequent phases; so evidently “innovation” in our recent descriptive phase contains “mythical potential” that cannot be inferred only from empirical studies.

“Innovation” is an academic construct created for the purposes of ‘evidence-based policy’ and imposed upon economic and social phenomena, still far from the way business think about change and improvements: respondents themselves use them relatively seldom. With respect to innovation surveys OECD expects that as they “gets repeated in many countries and the language of innovation becomes more widespread, the language and concept used in the Oslo Manual will achieve a material translation in the way business think about their own activities and achievements. Other academic constructs, for example in the management literature, appear to have gone through similar transitions but were supported by the interaction between business schools research and teaching”. However, “such a process is far from being complete in the field of innovation…” (OECD 2014a).

The benefits of innovation are much rather a question of faith than empirical proof. Even historical experience—the prolonged recession experienced by Japan, a country with an excellent record in innovation and the sudden surge in growth in China, a much less innovative country, history of the DDT and also the recent crisis connected with innovative financial products—have not cracked the steadfast conviction of the unconditional benefits generated by innovation. As with almost all social phenomena, innovations have their good and bad sides, depending on whom you refer to and the time frame you take into account.

Even a correlation between innovation and growth, as demonstrated by the economic literature, must be treated with caution, since the very concept of GDP has recently been subjected to critical discussion (Stiglitz et al. 2010; Karabell 2014).

The choice of indicators used in indices is often based on myths on the drivers of innovation. One of the myths concerns the role of patents in innovation and economic growth. For example, indices usually take into account patents as they are easy to count and are considered to have a strong link to innovation-led growth. However, patents play an ambiguous role in innovation and their importance varies greatly among the different sectors; they are often obtained for purely strategic reasons and many of them are of little value (Mazzucato 2014).

## Criticism of indices

As long as there are indices, there is a debate over their value and usefulness. Critics fall into two camps: some consider their errors to be treatable (“criticism in the family”), and some not (“criticism from outside”). Some disagree with the choice of partial indicators, while others think the problem is with the composite indicators.

Not advocating any of these options, I cite the seven strongest critical arguments raised during the debate:

- *First*, they are based on aggregates and averages.
- *Second*, they combine diverse and disparate indicators.
- *Third*, they assume the possibility of identifying the direction of causal relationships.
- *Fourth*, they are based on the possibility of determining the optimum.
- *Fifth*, they are based on data from the past with a view to help in deciding about the future.
- *Six*, they often cause undesirable side-effects.
- *Seven,* they use one measure to assess complex phenomena.

All this has consequences, which will be described shortly.

1. *Use of aggregates and averages* The majority of indices use the concept of the aggregates and averages as a reference point for comparing countries. With regard to statistical data used in indices, it is possible to apply what the nineteenth century Polish economist Zygmunt Heryng said of statistics: “economic statistics should present figures and facts in the form of properly grouped raw material [micro-data] and not, as has hitherto been the case, in the form of average combinations [macro-data].” Aggregated data hide the truth about economic phenomena and it is not known at a given time whether they “are actually a reflection of typical occurrences or merely a result of arithmetic and incapable of elucidating anything.” (Heryng 1896). Micro-data allows for a more precise identification of factors influencing country’s behaviour and a richer and more specific analysis.
  The same reservations must be made with regard to the average understood as an arithmetic mean, used in indices for assessing the position of countries.
  The importance of aggregates and averages is based on the conviction that people’s performance matches normal distribution (“bell curve”). However, a sample with an arithmetic average and an equal distribution above and below average rarely occurs (Taleb 2007). In particular, the majority of variables in science and innovation are characterised not by normal distribution but by asymmetric power law distribution (called Paretian).
2. *Diverse and disparate indicators* As a rule, indices are based on indicators describing extremely varied phenomena. Some indicators refer to clearly defined microeconomic facts (e.g. the number of enterprises receiving subsidies), while others refer to structural problems in the economy as a whole (inhabitants with higher education, employment in *high*-*tech* industries etc.). Some indicators are ‘structural’ in character, and usually only change over a long period of time, e.g. a society’s average level of education. Several indicators, such as expenditure on innovation of sales of new products, are dependent on changes in the business cycle. There are also indicators which are subject to short-term fluctuations in almost every country, e.g. indicators early stages of capital (*seed capital*) (Edquist and Zabala 2009). It is extremely difficult to combine such diverse indicator types in one index.
3. *Identifying causes* Indices assume the existence and possibility of identifying the direction of causal relationships, for their role is to indicate neglected elements or areas in need of such repair that can be reflected in indices. However, although determining the direction of causal relationships is prescribed by indices and the ‘logic of intervention’ of policy programs, this is very difficult in our reality, in which circular causality and non-linear effects are pre-eminent (Holland 1995). It is easy to mistake causes for symptoms. It is also easy to categorise one of the links in the chain of cyclical causality as a cause (e.g. mistaking investment in scientific research for a source of prosperity). Correlation is not causation: although innovation is generally considered to be a source of growth, the world’s leading technology countries foster innovation as a necessary long-term investment. Below the technological frontier innovation does not usually provide the same pay-off (Pessoa).
4. *Determining the optimum* The optimum level of a given indicator for a given territory is unknown, and perhaps unknowable. It is not always the case that an increase in an indicator (even the number of science and engineering graduates) unconditionally means an improvement in a determinant of innovation or the effectiveness of a system. It even isn’t true that ‘the more innovation, the better’. A system’s effectiveness (and this includes the innovation system) stems from the optimal balance of various factors. This balance varies for the different geographical regions being compared, as it depends on their characteristics (economic, historical, cultural). A feature of each system is limited resources, and allocating too many resources to the particular goals comes at the cost of the other. Bearing this in mind, the OECD emphasises that no single ideal combination of indicators exists for any policy. The effectiveness of indicators depends on the skill used in applying them in political processes. Furthermore, indicators are not a replacement for analyses aiming to establish correlations or causal relationships (OECD 2010).
5. *Conclusions about the future drawn from historical data* Indices are a reflection of the past, while phenomena described as “innovations” are in a constant state of flux. Indices are based on recognised indicators introduced at an earlier date in the majority of countries being compared. The implementation process for a new indicator takes many years. However, innovation practices change quickly and fundamentally, resulting in their being overlooked by previously established concepts and methods for measurement. Innovation indices are constantly posed the question of whether they are able to predict the future and indicate with accuracy how a country or region will develop, or whether they merely describe a current state, while true innovations remain a mystery, as do the time and place in which they will appear.
  Countries change their position not only by following the example set by others but also by utilising newly discovered (or recently emerging) opportunities. Indices do not take this fact into consideration for the simple reason that they are unable to. They are based on the assumption, often implicit but nevertheless largely shared, that current basis for growth lays the foundations for tomorrow’s prosperity (comp. Archibugi et al. 2009). This assumption may prove erroneous. Indices measure phenomena at the present time, using the latest data. They fail to consider events that have not taken place but could have taken place, variables hard to predict, which could have enormous impact on the future (Taleb 2007).They tend to be an imposition of short-term and often quite incorrect views onto the future. Innovation depends on many factors not included in indicators or indicators used in a particular set (Comp. Schibany and Streicher 2008).
6. *Unexpected negative consequences* There is often a lot of unexpected negative consequences of the hidden power of indices and indicators. “Those who intend to ground their choices of public policies also on statistical information, have to take care of distinguishing properly the indicators from the related phenomena. The policy aim is not, of course, to increase the value of the indicators, but the far more difficult problem of improving the economic and social conditions that the indicators are expected to capture. Scientific publications and patents, for example, are a means and not an end. But there is a danger that some policy-makers will concentrate on actions that have an effect on the indicator even when it is unclear if they also have an effect on the economic and social reality. For example, some governments distribute the resources devoted to academia on the basis of bibliometric indicators, giving an incentive to researchers to increase their publishable output rather than the knowledge generated. The outcome could be to transform scholars into scientific-article maximizers rather than into generators of knowledge” (Archibugi et al. 2009). Similarly, some less developed countries pay excessive attention to R&D and innovation indicators, while the “ultimate” goal of their public policy should be rather economic growth, prosperity, equality and justice, respect for the law, strategic and social security.
7. *One measure for assessing complex phenomena* There is no single measure that can reflect something as complex as our society. Any attempt to capture the state of the economy using too small a set of numbers can result in erroneous conclusions (Comp. Stiglitz et al. 2010).

In order to provide a platform to compare policy experiences and seek answers to common problems international organizations, such as OECD and European Union, arrange broad common conceptual frameworks, expressed in indicators, indices, information and policy platforms and analytical methods. These common conceptual frameworks are likely to give necessary basis for comparisons. The problem with these frameworks is that they are built on ideas developed in only one, the most developed part of the world, and that they often divert from seeking local idiosyncratic factors which may play decisive role in economic and social development. In indices, different countries are evaluated on the basis of one, universal criterion, which is created almost ‘made-to-measure’ for highly developed countries.

By way of necessity, indices take into consideration selected aspects of phenomena and overlook others, although it is difficult to foresee which aspects will prove to be of key importance for *upgrading* a particular region or country, and the success (or indeed failure) of each of these does not necessarily come down to the same factors. More often than not, they compare characteristics that are incommensurable (not on a common scale) using subjective evaluation of the level of their significance. Problematic aspects of phenomena (indicating the perspective of interest to us) transform into marks (e.g. the authors of indices select patents as indicators of innovation, deeming them to provide sound description of the subject, and then use the number of patents as a basis for judgements and evaluations of the state of innovation) (Ossowski 1962).

From the point of view of accuracy in relation to economies and societies, indices prove to be a better yardstick for some countries and regions than others, and are more effective at certain times than others. For instance, the *EU Innovation Indicator* is most effective in determining the dynamism of innovation in large, advanced countries with a highly developed manufacturing sector, such as France and Germany. It is less effective in describing the UK, with its large financial sector (overlooked in equations) and smaller southern countries, with their economies based on the sun, beaches and tourism.

Indices apply one yardstick to different economies. The level of innovation cannot be an absolute measure. It depends on economic structures, as particular sectors and groups in industrial production and services are not innovative to the same degree (OECD 2011). It is clear that innovations (as defined by the Oslo Manual 2005) are characteristic features of certain, advanced stages of economic development (“innovation-driven growth”, “prosperity-driven growth”), and are significant, though not to the degree seen at earlier stages (“investment-driven growth”). The type and character of innovation changes along with the transition to higher levels of development (e.g. not so much R&D as the purchase of machinery are characteristic of earlier stages of development, which may not lead to any measurable innovations in the Oslo Manual sense). As Archibugi et al. (2009) note, “we cannot expect the same causal relationships between technology and growth to have an identical impact on countries and regions that differ so greatly in their dimensions, income, infrastructure and human resources”.

The important defect of indices is that they are a relatively good measure of the success of economically developed countries, but constitute a poor basis for evaluating the progress of countries which are ‘playing catch-up’. Indices confirm what economic indicators and what can be seen, but are not a good instrument for assessing whether a country is getting closer to the economic leaders.

*Firstly,* indices rarely take account of the fact that during the catching-up process, at different levels of economic development measured by GDP per capita, the value of indicators change. For instance, in the initial stages of an industrial development, the levels of indicators such as primary and secondary schooling might rise rapidly, while indicators like R&D and innovation might rise insignificantly. Unusually high levels of indicators such as R&D expenditure as a proportion of GDP may be the hallmark of healthy developed economies or a sign of incorrect priorities have been chosen by a less developed economy. Holding a high position in an index is not always a sign that a country has made the right choices.

*Secondly,* indices rarely diversify evaluations according to the specific nature of a country. When “catching up”, every country strives to build on its initial advantages, although these differ from country to country and may not always be perceived by use of statistical indicators.

*Innovation Union Scoreboard*, though a good index, owes its position to the role it plays in EU policy. There is a danger that despite its imperfections, it will become one of the principal guidelines on the basis of the what were previously described as “self-fulfilling prophecies”.

In order to clearly define the shortcomings of indices, let us refer to an example from history. Nineteenth century Holland was no match for its southern neighbour Belgium in every sense related to industry. It had relatively few roads and a rail system which developed late and was less dense. In the mid-nineteenth century it possessed merely a tenth of Belgium’s total number of steam engines. Between 1869 to 1912 it had no patent law, which meant that no patents were issued during this period. It mined, processed and imported significantly less coal and steel. Had an index of industrialisation been produced in the nineteenth century, based on the assumption that industrialisation is an indication of future economic growth, Holland would have been ranked much lower than Belgium. And yet historical records of GDP per capita reveal that the Dutch standard of living was higher than that of Belgium, and was second only to Great Britain. This was due to the ‘engine of Holland’s growth’ not being industry, but trade, particularly international maritime trade. This explains the lower use of coal and steel. Holland’s excellent water transport system, based on canals, delayed the need for hard surfaced roads and railways. Assimilating technologies from abroad meant there was no need for the Dutch to come up with their own inventions. These facts misled historians, who had previously advanced the thesis of Holland’s economic decline in the nineteenth century (Wintle 2004). If historians get it wrong, even with the benefit of temporal distance, why should the creators of indices be expected to avoid error when designing indices whose evaluation possesses the power of forecasting, using the latest data to evaluate a country’s prospects? Applying one measuring tool to various mechanisms in the economy can lead us astray, and in the same way, applying various tools to different countries removes the possibility of drawing comparisons.

## A way out

If we are so critical of indicators, what should we do about it or what replace them with?

There are two solutions, one (moderate) in the short run, the second (radical) in the long run.

As concerns the short-run solution, three options are discussed.

First, to leave the situation as it is, but to strengthen the search for better indicators and better indices. Although their value has been exaggerated, indices have not yet said their final words. They remain a valuable source of information for policy-making. They should, however, be treated only as a point of departure for discussion and research and not as an irreversible verdict.

Second, abolishing indices and thus generating pressure to work on new econometric studies and policy studies, funded as a separate EU program.

Third, withdrawing the indices from centre stage in order to deprive policies of their real target and shifting them to the measurable components of the indices.

A radical long-term solution is associated with Big Data. A tool that will replace innovation indices will be based on big data. Big data have changed the way that we live, do business, manage organizations and carry out research. Big data are increasingly the fuel for and the driver of economic growth and form the basis for new research.^2^

With advent of “advanced data mining and analytics support, there seems to be fundamental changes that are occurring with the research questions we can ask, and the research methods we can apply” (Chang et al. 2014). The social and economic sciences which have traditionally relied on census statistics and surveys based on representative samples of populations can now make use of real-time data on the level of whole populations (OECD 2013).

I fully subscribe to the position that the statistics of the twentieth century were not designed for a new social and economic world of the twenty-first century and instead of “seeking new simple numbers to replace old simple numbers, economists need to tap into the power of the information age to figure out which questions need to be answered and to embrace new ways of answering them (…). To be useful, a new generation of indicators would have to answer particular, well-defined questions. But they cannot look like new versions of the old numbers. They cannot be one-size-fits-all generalizations. Instead of a few big averages, officials and ordinary people need a multiplicity of numbers that seek to answer a multitude of questions. In the era of ‘big data’, such an ambition is well within reach, thanks to powerful computing tools that can quickly process quantities of information that would have been unimaginable decades ago. In short, we do not need better leading indicators. We need bespoke indicators, tailored to the specific needs of governments, businesses, communities, and individuals-and we have the technology to provide them.” (Karabell 2014).^3^

So far it is not clear what kind of sound and non-trivial empirical correlations will emerge from future research. It is not clear what kind of data and analytical frameworks will replace innovation indices.

In order for this to be possible, changes are required to the three dimensions in which data are expressed: the dimensions of time, space and the characteristics of phenomena being described.

In terms of the *dimension oftime*, it is worth extending the scale and, where possible, conduct longitudinal research. Taking a broader view, embracing business cycles and even ‘long-term’ phenomena, creates a more sound basis for evaluation and prediction. For instance, one interesting analysis would be that of the second series of data on science and economic variables (1960–2012) based on OECD, World Bank and UNESCO archives, the aim of which would be to work out structures of catching-up for those states experiencing economic growth in the course of the last 50 years.

In terms of the *dimension of space*, one could attempt to look at this as a collection of places and relations using the Geographical Information System (GIS). The reason for its importance is that, as the fundamental laws of geography state, “[a]ll things are related, but nearby things are more related than distant things” (Tobler 1970). “Without space, we have only one place, and it can tell us only one story; with space, we have multiple places and each of these can behave differently” (Knowles 2008). Research based on the GIS is expensive, laborious and time-consuming, nonetheless when related to innovation and economic growth, these are both interesting and cost-effective.

In terms of the *characteristics*, one interesting avenue to explore would be a broader consideration of the specifics nature of sectors of industry, technological groups, scientific disciplines and extending data on R&D and innovation to include culture, education and creativity.

## Instead of a conclusion

If we are so critical of indicators, what should we do about it or what replace them with?

This is a difficult question to answer as no ideal recipe for successful research exists. Original findings are the result of numerous attempts and explorations, and emerge more often from the mists of uncertainty than from algorithms provided by indices. The search for truth is better expressed by Heidegger’s metaphors of “wandering along a forest path” with neither a clear destination nor direction, in the hope of spotting a “clearing”, rare moments of enlightened observation than by the idea that this search means adhering to methods imposed and defined a priori, which predetermine what we will find (comp. Buczyńska-Garewicz 2008). Nassim Nicholas Taleb concept of “stochastic tinkering”, namely bottom-up experimenting research, based on small steps, randomness and serendipity, is also more appropriate (Taleb 2007). It would be better if researchers were to remain in the state philosophers define as “aporia”, a state of cognitive uncertainty, doubt, surprise, and indecision, rather than claim that they have discovered the best method of evaluation, one which can be encapsulated in an index.

One indication of wisdom is the skill of noticing the many-faceted nature of phenomena. From this point of view the domination of one particular framework has the advantage of acting as a catalyst for ideas, but the disadvantage of pinning an idea in a trap. Indices are as good when used as “a matrix for presenting problems” and abandoned once they have fulfilled their purpose.

The propositions concerning the lack of neutrality in the language of cognition and the absence of a single, definitive perspective lead us to conclude that in order to understand phenomena, it is necessary to abandon one framework and acknowledge that “several may be correct at the same time”, as “truth has many forms and many foundations”. Each framework is the result of a series of choices, which, though well justified, denote a rejection of certain paths, ignoring certain possibilities which might later prove to be of value (Judt and Snyder 2012). An index packs each and every problem into one Procrustean bed, thus gaining coherence and clear-cut nature at the expense of everything that does not fit the core principle. This provides fuel for the originators of new ideas, who indicate deficiencies and inconsistencies in existing indices and instead offer their own.

It also transpires that a particular framework, e.g. the *European Union Innovation Scoreboard*, forces others aside, on the basis of its being economical and wide-ranging, having official status, a catchy name, being fashionable or appearing to all as ‘self-evident’. In short, such a framework appears to those who are not well-informed as “maps” which map out all the features of an area in a neutral and objective way.

However, a map is not objective. Like an index, it is a tool of communication, persuasion and authority. It is the sum total of choices, such as the choice of scale, grid coordinates and symbols, as well as the author’s standpoint, fields of interest and own interests. Like an index, each separate map is one of an infinite number of maps which can be drawn up using the same raw data. Like an index, a map conceals its subjectivity behind its appearance of objectivity. A map showing the size and shape of a territory is merely a fictional paradox, while a useful, precise and genuine map still “lies”. The “lie” of maps is unavoidable (Wood 1992). The problem arises when the map is “taken as the territory”, when on the strength of an official decision, a map is arbitrarily acknowledged as the source of truth, or when the existence of other maps is forgotten.

Because “map is not territory” indices are not congruent with the phenomena that they describe.

The search for new tools for measuring innovation can be enriched by an analysis of non-innovation. In *Die Probleme der Geschichtsphilosophie*, Simmel noticed that “phenomena are so numerous, varied and complicated, and move in such complex whirling motions that getting one’s bearings is only possible by placing a frequently perceived fact at the centre of their world view…“Although this is possible only through bending and smashing reality, “it holds the guiding thread in order not to stray into the vortex of phenomena” (Simmel 1892). Research on innovation, and especially innovation indices, places innovation at the centre of the human world. However, in the real world innovation is rarely as important as in policy rhetoric and innovation indices. Research on non-innovation (going much further than studies on non-innovative firms and different from sociological studies on tradition) might restore a proper balance in understanding change and duration, as it is wrong to believe that studying the phenomenon of innovation alone leads us to understand the phenomenon of duration.

It is also worth considering a greater number of variables than in indices; it is worth expressing them in their mutual links and analysing the subject on a variety on levels and from different perspectives. It is better to tackle specific problems, e.g. the problem of sources of disparities in the development paths of different countries and regions, rather than search for one broad formula for evaluating such diverse units.

In his famous essay “The Priest and the Jester”, Leszek Kołakowski (2003) presents intellectual life as the dichotomy between philosophy as the guardian of the absolute and philosophy as questioning of the absolute. Where the priest is honourable and guards tradition, the jester is impertinent and questions every certainty. The priest praises that which is ultimate and closed, the jester that which is what is open, paradoxical, diverse. Conflict between the priest and jester is unavoidable, for each represents virtues in every cognitive activity: without the priest there can be no coherent objectives, without the jester no inquisitiveness (Torgerson 1992).

As Kołakowski stated, in the court of the king the priests usually outnumber the jesters, hence it is all the more true that the jester is worth listening to.

The jester’s message in discussions on indices can be summed up as follows: Right from the start and quite arbitrarily indices impose definitions and a sense of what they are seeking, which robs them of the possibility of finding something quite new (comp. Buczynska-Garewicz 2008). The progress in science and innovation policy studies depends on a diversity of issues, approaches and perspectives. If that is the case, maintaining thematic and methodological variety may be more important than creating coherent and closed analytical tools, i.e. officially supported indices (comp. Gläser et al. 2002). Universally applied as they are, indices shape the future. They unconsciously eliminate the possibility of un-promoted mental and cognitive phenomena. They only foster what is already contained in our imagination at present and in a significant way restrict our future by eliminating other possibilities from the course of evolution.

## Footnotes

1 The article express the opinions of the author and not of the Ministry of Science and Higher Education. I would like to express my deep gratitude to Ms. Paulina Zadura-Lichota from the Polish Agency for Enterprise Development for inspiring subject matter, Prof. Hanna Buczynska-Garewicz, Prof. Benoǐt Godin, Prof. Loet Leydesdorff and Prof. Jacek Guliński for comments and encouragement and the Salzburg Group for the opportunity to discuss ideas. In particular I would thank the anonymous reviewer for his tireless effort to improve the text.

2 Six categories of data are identified: data stemming from the transactions of government; data describing official registration or licensing requirements; commercial transactions made by individuals and organizations; Internet data, deriving from search and social networking activities; tracking data, monitoring the movement of individuals or physical objects subject to movement by humans; and, finally, image data, particularly aerial and satellite images but including land-based video images. Expansion of data is largely driven by the “increasing ubiquity of broadband access and the proliferation of smart devices and smart ICT applications such as smart meters, smart grids and smart transport based on sensor networks and machine-to-machine (M2M) communication” (OECD 2013).

3 Some of these postulates were put forward earlier, comp. Lepori et al. (2011).
